# Supplementary material for: Understanding the Problem of Access to Public Health Insurance Schemes among Cross-Border Migrants in Thailand through Systems Thinking
Source: Int J Environ Res Public Health. 2020 Jul 15;17(14):5113. doi: 10.3390/ijerph17145113 (PMC7400460; doi:10.3390/ijerph17145113)
Supplement: Supplementary file 1 [file ijerph-17-05113-s001.pdf]

**Supplementary materials**

**Table S1** Characteristics of participants engaging in the 1ST and 2ND GMBs

| Meeting | Coding | Sex | Age | Career | Career level | Organization | Sector | Work experience |
|---|---|---|---|---|---|---|---|---|
| 1, 2 | SD1 | 2 | 53 | Project manager | senior | National Health Security Office (NHSO) | Health | More than 30 years |
| 1, 2 | SD2 | 2 | 54 | Research project Manager | senior | HSRI | Health | More than 30 years |
| 1 | SD3 | 2 | 51 | Senior researcher | senior | Disease control, Ministry of Public Health, Thailand | Health | Less than 10 years |
| 1 | SD4 | 2 | 54 | Senior nurse | senior | Department of Health, Ministry of Public Health, Thailand | Health | More than 30 years |
| 1 | SD5 | 1 | 54 | Project manager | senior | NGO A | Civil society | More than 30 years |
| 1, 2 | SD6 | 1 | 43 | Project coordinator | coordinator | NGO B | Civil society | More than 30 years |
| 1 | SD7 | 1 | 31 | Lawyer | middle | NGO C | Civil society | Less than 10 years |
| 1, 2 | SD8 | 2 | 29 | Volunteer | middle | NGO D | Civil society | Less than 10 years |
| 1 | SD9 | 1 | 30 | Researcher | middle | Ministry of Labour of Thailand | Non-health | Less than 10 years |

| Meeting | Coding | Sex | Age | Career | Career level | Organization | Sector | Work experience |
|---|---|---|---|---|---|---|---|---|
| 1 | SD10 | 2 | 34 | Researcher | middle | Health Intervention and Technology Assessment Program (HITAP), Ministry of Public Health, Thailand | Health | Less than 10 years |
| 1 | SD11 | 1 | 29 | Research assistant | coordinator | Health Intervention and Technology Assessment Program (HITAP ), Ministry of Public Health, Thailand | Health | Less than 10 years |
| 2 | SD12 | 1 | 42 | assistant deputy director of department of finance, MOPH | senior | Ministry of Public Health of Thailand | Health | Less than 10 years |
| 2 | SD13 | 2 | 53 | Research project Manager | senior | National Health Security Office (NHSO) | Health | More than 30 years |
| 2 | SD14 | 2 | 37 | Lecturer | middle | Faculty of Law, Naresuan University | Academic | 10-19 years |
| 2 | SD15 | 2 | 27 | Lawyer | middle | Bangkok clinic, Thammasat University | Civil society | Less than 10 years |

| Meeting | Coding | Sex | Age | Career | Career level | Organization | Sector | Work experience |
|---|---|---|---|---|---|---|---|---|
| 2 | SD16 | 2 | 59 | Deputy director of monitoring and evaluation centre for human's right | senior | National human rights commission of Thailand | Civil society | More than 30 years |
| 2 | SD17 | 2 | 37 | Researcher | senior | Health Systems Research Institute (HSRI) | Health | 10-19 years |
| 1 | SD18 | 1 | 31 | Lawyer | middle | NGO C | Civil society | Less than 10 years |
| 1 | SD19 | 2 | 29 | Volunteer | middle | NGO D | Civil society | Less than 10 years |
| 1 | SD20 | 2 | 53 | Project manager | senior | National Health Security Office (NHSO) | Health | More than 30 years |

Note: sex 1= Male, 2 = Female
